# Perceived Noise Levels in Intensive Care Units and Their Self-Reported Adverse Effects on Nurses in Saudi Arabia: A Cross-Sectional Study

**DOI:** 10.3390/nursrep16090342

**Published:** 2026-09-19

**Authors:** Nabat Almalki, Atheer Asiri, Lama Alkhaldi, Huda Majrashi, Abeer Almotairi, Amal Alfifi, Hind Althobaiti, Badriah Althubaiti, Jawharah Alhufayyan, Bushra Alshammari, Mawahib Almalki

**Affiliations:** 1 Nursing Department, Prince Sultan Military College of Health Sciences, Dhahran 34313, Saudi Arabia; 2 Al Kharj Military Industries Corporation Hospital, Al-Kharj 16274, Saudi Arabia; ath220125@gmail.com; 3 Prince Mansour Military Hospital, Taif 26526, Saudi Arabia; lama1383@hotmail.com (L.A.); abeeeer12334@gmail.com (A.A.); 4 Prince Sultan Military Medical City, Riyadh 12233, Saudi Arabia; dooshmy@gmail.com; 5 Alhada Armed Forces Hospital, Taif 26792, Saudi Arabia; cococacake2013@hotmail.com (A.A.); hm.1440@hotmail.com (H.A.); lood.2014@hotmail.com (B.A.); 6 King Fahad Armed Forces Hospital, Jeddah 23311, Saudi Arabia; jojoam2004@hotmail.com; 7 Medical Surgical Nursing Department, College of Nursing, University of Hail, Hail 2440, Saudi Arabia; bu.alshammari@uoh.edu.sa; 8 School of Nursing and Midwifery, Queen’s University Belfast, Medical Biology Centre, Belfast BT9 7BL, UK

**Keywords:** intensive care unit, ICU noise, perceived noise, nurses, occupational stress, alarm fatigue, Saudi Arabia

## Abstract

**Background/Objectives**: Intensive care units (ICUs) are acoustically demanding environments in which nurses are continuously exposed to alarms, medical equipment, and staff activity. This study assessed ICU nurses’ perceived internal and external noise levels and their perceived noise-related adverse effects, and examined whether these perceptions differed according to demographic and work-related characteristics. **Methods**: A cross-sectional design was used with 244 ICU nurses in Saudi Arabia. Data were collected using a structured questionnaire adapted to assess perceived internal and external ICU noise and four effect domains (subjective, emotional, physiological, and work performance). **Results**: Perceived ICU noise was moderate (internal 64.68; external 64.51). Effect-domain means were 50.95 (subjective), 54.67 (emotional), 54.74 (physiological), and 51.28 (work performance). Female nurses scored significantly higher than male nurses for internal and external noise and for subjective, emotional, and physiological effects (all *p* < 0.05). Staff nurses, rotating-shift nurses, and nurses with <5 years of ICU experience reported higher effects in several domains; significant differences were also found by age group, marital status, and work-shift pattern. In multivariable models adjusting for all characteristics simultaneously, staff-nurse role, female gender and rotating shift remained independently associated with higher scores, whereas age group and years of ICU experience did not. **Conclusions**: ICU noise was perceived as a significant occupational stressor, with certain nurse subgroups reporting greater adverse effects. Healthcare organizations should prioritize comprehensive noise-reduction policies, effective alarm management, and targeted support strategies to mitigate noise-related impacts on nurses and improve the ICU work environment.

## 1. Introduction

Intensive care units (ICUs) are among the most complex environments in modern healthcare, where critically ill patients require continuous monitoring, advanced technology, and rapid clinical decisions. Nurses are central to maintaining patient safety in these settings while working under high workload, time pressure, and multiple environmental stressors [1]. Among these stressors, noise has emerged as a persistent concern: ICUs are characterised by elevated sound from monitoring devices, alarms, ventilators, staff activity, and routine care procedures [2]. Recorded ICU sound levels frequently exceed international recommendations—for example, values of 60.5–94.6 dBA have been reported during the day [3], and one cohort found that less than 1% of monitored time fell below guideline limits [2].

The World Health Organization recommends that hospital noise should not exceed 35 dBA during the day and 30 dBA at night [4,5]. Sustained exposure above these thresholds has been linked to disrupted patient sleep and physiological instability [3], and, for nurses, to increased stress, fatigue, reduced concentration, and impaired performance [6,7]. Alarm-related noise is a particularly important contributor: nurses may face 150–400 alarms per patient per day [8], of which 85–99% may be non-actionable [9], driving the phenomenon of alarm fatigue and desensitisation to critical alerts [10]. Reported noise effects on nurses span emotional, physiological, and performance domains [6,11], and are associated with reduced quality of nursing work life [12] and with alarm fatigue itself [13,14]. Evidence from Saudi Arabia further indicates that noise from alarms, equipment, and night-time activity disrupts patients and staff alike [15]. In this study, ICU noise sources were classified as internal or external, consistent with Kooshanfar et al. [6]. Internal noise refers to sound originating inside the ICU, such as alarms, medical equipment, staff conversations, and routine patient-care activities. External noise refers to sound originating from other areas within the hospital such as hospital renovation activities [6].

Although ICU noise has been widely studied in relation to patient comfort, its perceived adverse effects on nurses remain comparatively under-examined, especially within Saudi healthcare settings where organisational structure, staffing, and technology use may shape how noise is experienced [6,7]. The present study therefore aimed to (i) assess ICU nurses’ perceived internal and external noise levels; (ii) assess perceived noise-related adverse effects across subjective, emotional, physiological, and work-performance domains; and (iii) examine whether these perceptions differed by gender, marital status, age group, job title, years of ICU experience, and work-shift pattern.

## 2. Materials and Methods

### 2.1. Study Design and Setting

A descriptive cross-sectional design was used to capture ICU nurses’ perceptions of noise sources, discomfort, stress, and self-reported performance impact at a single point in time. The study was conducted across twelve ICUs in four tertiary military hospitals located in Riyadh, Taif, Jeddah, and Al-Kharj, Saudi Arabia. These included cardiac, general, medical, neonatal, pediatric, and surgical ICUs. Approximately 400 nurses were working across these units.

### 2.2. Sample and Sample-Size Estimation

The target population was registered nurses with ICU experience. A convenience sampling method was used. Inclusion criteria were being a registered nurse with at least one year of ICU experience and voluntary willingness to participate. The minimum sample size was estimated using the single-proportion formula, *n* = *Z*^2^·*p*(1 − *p*)/*d*^2^, with *Z* = 1.96, *p* = 0.50 (maximum variability), and *d* = 0.08, yielding a minimum sample size of 150 participants. Exclusion criteria were nurses with less than one year of ICU experience, nurses not currently assigned to an ICU at the time of data collection (e.g., those on extended leave or redeployed to non-critical-care areas).

### 2.3. Instrument

Data were collected using the structured questionnaire previously developed and validated by Kooshanfar et al. [6], which was administered in its original language. It comprise three sections: (1) demographic and job-related characteristics; (2) perceived internal and external noise sources rated on a three-point Likert scale (1 = little, 2 = moderate, 3 = much); and (3) perceived noise-related effects across four domains—subjective, emotional, physiological, and work performance—rated on a five-point Likert scale (1 = never to 5 = always). The instrument showed acceptable internal consistency (Cronbach’s α = 0.852). Although the questionnaire demonstrated acceptable internal consistency in the present sample, it has not undergone comprehensive psychometric validation among ICU nurses in Saudi Arabia. For analysis and reporting, each subscale was transformed to a 0–100 score, where higher values indicate greater perceived noise or a greater perceived effect. Specifically, each subscale score was calculated as: transformed score = (mean item score/maximum possible score) × 100, so that the three-point noise items and five-point effect items were expressed on a common metric. Because this transformation retains the scale floor, the attainable range is 33.3–100 for the noise subscales and 20–100 for the effect subscales; scores should therefore be read as relative positions on a common metric rather than as percentages of a true zero point.

### 2.4. Data Collection and Ethics

Ethical approval was obtained from the Institutional Review Board of Prince Sultan Military College of Health Sciences (PSMCHS) (IRB-2026-NUR-057; 13 April 2026). Participants received a clear explanation of the study’s objectives, the voluntary nature of participation, and their right to withdraw at any time. Informed consent was obtained electronically at the start of the questionnaire. No personal identifiers were collected, and all data were stored securely and used exclusively for research purposes.

The questionnaire was distributed electronically in April 2026 through professional nursing networks. Eligible ICU nurses received a link to the online questionnaire, which included information about the study and an electronic consent form (see the questionnaire in Supplementary Materials).

### 2.5. Statistical Analysis

Data were analysed using IBM SPSS Statistics version 29.0 (IBM Corp., Armonk, NY, USA). Frequencies and percentages summarised categorical characteristics; means and standard deviations described perceived noise and effect scores. For between-group comparisons, independent-samples *t*-tests were used for two-group variables, while one-way ANOVA (F-test) and Kruskal–Wallis tests were used for variables with more than two groups, selected according to distributional assumptions for each variable. A two-sided *p* < 0.05 was considered statistically significant. To account for possible interrelationships among the predictors, multivariable linear regression models were additionally fitted for each noise and effect domain, entering gender, age group, marital status, job title, years of ICU experience, and work-shift pattern simultaneously as independent variables. Categorical predictors were entered as indicator variables with the following reference categories: male, 18–25 years, single, charge nurse, <5 years of ICU experience and night shift. Unstandardised (B) and standardised (β) coefficients with 95% confidence intervals were estimated, and variance inflation factors were examined to exclude multicollinearity. Because Breusch–Pagan tests indicated non-constant residual variance in five of the six models, heteroscedasticity-consistent (HC3) standard errors were used for all inference, and the joint contribution of each multi-category predictor was assessed with a Wald test across its indicator variables. The bivariate comparisons are retained for transparency and are interpreted alongside the adjusted models.

## 3. Results

### 3.1. Participant Characteristics

A total of 244 nurses participated in the study, with a response rate of 61%. The participants were predominantly female (170; 69.7%). The most common age group was 26–35 years (139; 57.0%); most were married (129; 52.9%) and held a bachelor’s degree (151; 61.9%). Staff nurses formed the largest job-title group (101; 41.4%), most worked in a general ICU (77; 33.0%), and 119 (48.8%) had 5–13 years of ICU experience. Rotating shifts were most common (112; 45.9%), and 130 nurses (53.3%) reported less than 10 h of monthly overtime (Table 1).

### 3.2. Between-Group Comparisons of Perceived Noise and Effect Scores

Female nurses reported significantly higher scores than male nurses for internal ICU noise (66.11 vs. 61.38; *p* = 0.01), external ICU noise (66.14 vs. 60.76; *p* = 0.003), subjective effects (53.72 vs. 44.59; *p* < 0.001), emotional effects (56.64 vs. 50.16; *p* < 0.001), and physiological effects (56.56 vs. 50.54; *p* = 0.002). No significant difference was found for work performance (*p* = 0.14). Unadjusted comparisons across all six demographic and work-related characteristics are reported together in Table 2; post-hoc results are given in the table note. (Table 2).

Married participants reported slightly higher work-performance effect scores than single participants (52.93 vs. 49.43; mean difference = 3.50 points). Although this difference met the conventional threshold before adjustment (*t* = 2.00, unadjusted *p* = 0.04), it did not remain statistically significant after Holm–Bonferroni correction for the six comparisons (*p* adjusted = 0.24). Therefore, this finding should be regarded as exploratory and interpreted cautiously. No statistically significant differences by marital status were observed after adjustment.

Age group was significantly associated with emotional effects (*p* = 0.047) and physiological effects (*p* = 0.022). Nurses aged ≥ 46 years reported the highest emotional (66.29) and physiological (60.00) means, although this group was small (*n* = 7). Post-hoc comparisons (Dunn’s test with Bonferroni adjustment) did not identify statistically significant pairwise differences between age groups, so this age-related pattern should be interpreted with caution. No significant differences were found for internal noise, external noise, subjective effects, or work performance (Table 2).

Job title showed the strongest and most consistent differences. Significant differences were found for internal noise (*p* < 0.001), external noise (*p* = 0.005), subjective effects (*p* < 0.001), emotional effects (*p* = 0.03), and physiological effects (*p* < 0.001). Staff nurses reported the highest means in every significant domain, including internal noise (68.77), external noise (67.73), subjective (58.42), emotional (57.50), and physiological (59.84) effects. Work performance did not differ significantly (*p* = 0.90) (Table 2). Post-hoc Tukey’s HSD comparisons confirmed that staff nurses scored significantly higher than both charge nurses and head nurses for internal noise and for subjective, emotional, and physiological effects, and significantly higher than charge nurses for external noise (all adjusted *p* < 0.05).

Years of ICU experience was significantly associated with subjective effects (*p* = 0.04) and physiological effects (*p* = 0.012). Nurses with <5 years of experience reported the highest subjective (53.77) and physiological (57.69) means. No significant differences were found for internal noise, external noise, emotional effects, or work performance (Table 2). Post-hoc comparisons (Dunn’s test with Bonferroni adjustment) showed that nurses with <5 years of ICU experience scored significantly higher than those with 5–13 years for subjective and physiological effects (adjusted *p* < 0.05).

Work-shift pattern was significantly associated with internal noise (*p* = 0.03), external noise (*p* = 0.03), subjective effects (*p* < 0.001), emotional effects (*p* = 0.015), and physiological effects (*p* < 0.001). Morning-regular-shift nurses reported the highest perceived internal (67.80) and external (68.52) noise, whereas rotating-shift nurses reported the highest subjective (56.14), emotional (57.21), and physiological (58.18) effects. Work performance did not differ significantly (*p* = 0.08) (Table 2). Post-hoc comparisons (Dunn’s test with Bonferroni adjustment) indicated that rotating-shift nurses scored significantly higher than night-shift nurses for subjective, emotional, and physiological effects (adjusted *p* < 0.05).

### 3.3. Multivariable Regression Analysis

In the adjusted multivariable models, several of the associations observed in the bivariate analyses were attenuated once the remaining characteristics were held constant. Job title remained independently associated with perceived internal noise (staff nurses vs. charge nurses, B = 6.63, 95% CI 2.04 to 11.23; *p* = 0.005) and with subjective (B = 8.35, 95% CI 3.10 to 13.60; *p* = 0.002) and physiological effects (B = 5.89, 95% CI 1.01 to 10.76; *p* = 0.018). Female gender remained independently associated with higher external noise (B = 3.62, 95% CI 0.26 to 6.98; *p* = 0.035) and with higher subjective (B = 4.69, 95% CI 1.24 to 8.14; *p* = 0.008) and emotional effects (B = 4.72, 95% CI 1.19 to 8.25; *p* = 0.009), but not with internal noise or physiological effects. Relative to night-shift nurses, rotating-shift nurses reported higher subjective (B = 5.97; *p* = 0.013), emotional (B = 4.30; *p* = 0.047), physiological (B = 4.72; *p* = 0.028), and work-performance effects (B = 5.65; *p* = 0.007), although work-shift pattern did not reach significance as a block in any model. Married participants reported higher physiological effects than single participants (B = 4.11, 95% CI 0.19 to 8.02; *p* = 0.040). Neither age group nor years of ICU experience was independently associated with any outcome. The models accounted for 7.4–24.3% of the variance (adjusted R^2^ 0.026–0.204), and the overall model for work performance was not statistically significant (F(12, 231) = 1.63; *p* = 0.083). Variance inflation factors ranged from 1.14 to 2.95, indicating no problematic multicollinearity. Full model output is presented in Table 3.

## 4. Discussion

This study assessed ICU nurses’ perceived noise levels and noise-related adverse effects and examined how they varied by demographic and work-related characteristics. Overall, perceived internal and external ICU noise were moderate, and all four effect domains showed meaningful perceived impact. Gender was the clearest correlate of perceived noise: female nurses reported significantly higher internal and external noise and higher subjective, emotional, and physiological effects, but not work performance. In the adjusted models this gender difference persisted for external noise and for subjective and emotional effects, but not for internal noise or physiological effects. This aligns with reports that noise exposure affects multiple facets of nurses’ well-being—stress, irritability, fatigue, and physiological strain [6,11]. A plausible biological contributor to this gender gap is that women show, on average, greater cochlear sensitivity than men across diverse populations, which could lower the threshold at which ambient ICU sound is perceived as intrusive [16]. The absence of a gender difference in perceived work performance may indicate that nurses sustain clinical duties despite noise-related discomfort.

Job title produced the strongest differences, with staff nurses scoring highest across all significant domains. This is expected, as staff nurses remain closest to bedside alarms, procedures, and routine activity; monitoring alarms and ventilators are known to dominate internal ICU noise (≈61.5% of sources) [6], and perceived noise has been linked to nurses’ health and performance [11]. In unadjusted comparisons, less-experienced nurses (<5 years) reported higher subjective and physiological effects, consistent with the demands of adapting to a complex, alarm-rich technological environment and with evidence that mental fatigue raises error likelihood [1]. Work-shift pattern also mattered: morning-regular shifts were associated with higher perceived noise (likely reflecting daytime clinical activity), whereas rotating shifts were linked to greater perceived effects, echoing associations between workplace noise, sleep quality, and quality of nursing work life [12]. After simultaneous adjustment, however, the experience gradient was no longer significant, whereas the staff-nurse and rotating-shift differences persisted (Table 3), suggesting that role and shift pattern rather than experience itself explain much of the observed variation.

Age was associated with emotional and physiological effects in unadjusted analysis, with the highest means among nurses aged ≥ 46 years; however, this subgroup was small (*n* = 7) and its estimates were imprecise, and the association did not persist after adjustment. Marital status showed a single significant unadjusted difference (work performance), and married participants reported higher physiological effects in the adjusted model.

Collectively, the pattern of significant emotional and physiological effects—particularly among female, staff and rotating-shift nurses—supports the view that ICU noise is an occupational and clinical issue rather than a mere inconvenience and is closely tied to the broader problem of alarm fatigue [8,9,10].

These findings carry practical implications for ICU noise-management strategies. Structured “quiet time” protocols, involving scheduled reductions in conversation, dimmed lighting, and clustered care activities during defined periods, have been associated, across several ICU and ward settings, with lower peak noise levels and with reduced nurse-reported stress, alongside variable but generally favourable effects on patient sleep [17]. Combined with the alarm-management and staffing considerations raised by earlier ICU noise research [6,9], such protocols may be particularly valuable for the staff-nurse and rotating-shift subgroups identified in the present study as bearing a disproportionate share of the perceived noise burden.

### Strengths and Limitations

Strengths include using of validated instrument with good internal consistency, supported by expert review and pilot testing. The study also addresses a context of Saudi ICU nurses that remains underexplored in the noise perception literature, adding valuable evidence to a limited evidence base. Nonetheless, several limitations should be noted. The cross-sectional design precludes causal inference, and reliance on self-report captures perceived rather than objectively measured noise. Convenience sampling may also limit generalisability. Future research should combine perceived measures with objective dosimetry and replicate these multivariable models in probability samples. Although the questionnaire demonstrated good internal consistency and underwent expert review and pilot testing, it has not received comprehensive psychometric validation among Saudi ICU nurses, so its construct validity and temporal stability in this context remain to be established. Future studies should undertake a full validation, including factor analysis and test–retest reliability.

## 5. Conclusions

This study demonstrates that ICU noise is not merely an environmental nuisance but a significant occupational stressor with measurable subjective, emotional, physiological, and work performance consequences for nurses. The finding that female nurses, staff nurses, and those working rotating shifts independently reported higher adverse effects, even after adjusting for other characteristics, suggests that noise burden is not distributed evenly across the nursing workforce. This pattern may reflect differences in shift related fatigue, role related proximity to alarm sources, and cumulative exposure, rather than experience or age alone. These results indicate that ICU noise management should move beyond generic environmental interventions toward targeted, subgroup sensitive strategies. Structured quiet time protocols, refined alarm management systems, and adequate staffing on rotating shifts may help reduce both peak noise exposure and the disproportionate strain experienced by staff nurses and rotating shift personnel. Future research should evaluate the effectiveness of such targeted interventions using longitudinal or interventional designs and should examine whether reducing perceived noise burden translates into measurable improvements in nurse well-being, retention, and patient safety outcomes. Addressing ICU noise as a modifiable occupational hazard, rather than an unavoidable feature of critical care, represents an important step toward safer and more sustainable ICU work environments.

## Figures and Tables

**Table 1:** Demographic and work-related characteristics of participants (*n* = 244).

Characteristic	Category	*n*	%
Gender	Male	74	30.3
	Female	170	69.7
Age group (years)	18–25	31	12.7
	26–35	139	57.0
	36–45	67	27.5
	≥46	7	2.9
Marital status	Single	115	47.1
	Married	129	52.9
Educational level	Diploma	54	22.1
	Bachelor	151	61.9
	Master	37	15.2
	Ph.D.	2	0.8
ICU type ^a^	Cardiac ICU	47	20.2
	General ICU	77	33.0
	Medical ICU	14	6.0
	Neonatal ICU	55	23.6
	Pediatric ICU	36	15.5
	Surgical ICU	4	1.7
Job title	Charge nurse	84	34.4
	Head nurse	59	24.2
	Nurse	101	41.4
Years of ICU experience	<5	97	39.8
	5–13	119	48.8
	>13	28	11.5
Work-shift pattern	Day shift	37	15.2
	Morning regular shift	16	6.6
	Night shift	79	32.4
	Rotating (both)	112	45.9
Average monthly overtime	<10 h	130	53.3
	>10 h	114	46.7

Note: *n*, number of participants; %, percentage; ICU, intensive care unit; ^a^ ICU type was reported by 233 participants (11 did not specify); percentages for this variable are based on *n* = 233.

**Table 2:** Perceived noise and effect scores by demographic and work-related characteristics (*n* = 244).

Characteristic	*n*	Internal Noise	External Noise	Subjective	Emotional	Physiological		Work Performance
Gender		*t* = 2.59; *p* = 0.010 *		*t* = 2.97; *p* = 0.003 *	*t* = 4.43; *p* ≤ 0.001 *	*t* = 3.37; *p* ≤ 0.001 *	*t* = 3.15; *p* = 0.002 *	*t* = 1.49; *p* = 0.137
Male	74	61.38 (10.72)	60.76 (9.28)	44.59 (9.81)	50.16 (10.86)	50.54 (9.88)	49.30 (10.70)	
Female	170	66.11 (14.00)	66.14 (14.32)	53.72 (16.46)	56.64 (14.87)	56.56 (15.09)	52.14 (14.79)	
Age group (years)		H = 4.35; *p* = 0.226	H = 4.17; *p* = 0.244	H = 5.78; *p* = 0.123	H = 7.95; *p* = 0.047 *	H = 9.62; *p* = 0.022 *	H = 4.76; *p* = 0.190	
18–25	31	61.39 (12.62)	60.69 (11.54)	47.87 (11.71)	53.16 (12.23)	54.19 (11.12)	48.77 (10.30)	
26–35	139	65.84 (13.86)	65.52 (14.57)	53.27 (16.96)	55.48 (15.90)	56.46 (15.19)	50.85 (14.81)	
36–45	67	63.23 (11.70)	63.74 (10.82)	47.22 (11.19)	52.48 (10.08)	50.87 (11.77)	52.36 (11.73)	
≥46	7	70.13 (15.69)	68.78 (10.44)	54.29 (22.25)	66.29 (10.29)	60.00 (13.86)	60.57 (19.52)	
Marital status		*t* = 1.49; *p* = 0.138	*t* = 1.88; *p* = 0.061	*t* = 1.13; *p* = 0.258	*t* = 1.23; *p* = 0.218	*t* = 1.20; *p* = 0.231	*t* = 2.00; *p* = 0.046 *	
Single	115	63.35 (13.48)	62.83 (13.15)	49.77 (15.48)	53.50 (14.55)	53.60 (13.85)	49.43 (14.01)	
Married	129	65.87 (12.98)		66.01 (13.14)	52.00 (15.18)	55.72 (13.61)	55.75 (14.06)	52.93 (13.29)
Job title		F = 9.00; *p* ≤ 0.001 *		F = 5.52; *p* = 0.005 *	F = 24.43; *p* ≤ 0.001 *	F = 3.56; *p* = 0.030 *	F = 13.21; *p* ≤ 0.001 *	F = 0.11; *p* = 0.899
Charge nurse	84	61.15 (10.08)		61.64 (9.89)	46.00 (12.76)	52.67 (12.40)	50.14 (11.41)	51.05 (11.61)
Head nurse	59	62.71 (10.89)		63.09 (10.70)	45.22 (10.54)	52.68 (12.63)	52.54 (12.24)	52.00 (13.64)
Staff nurse	101	68.77 (15.62)		67.73 (16.07)	58.42 (16.59)	57.50 (15.74)	59.84 (15.26)	51.05 (15.40)
Years of ICU experience		H = 2.33; *p* = 0.311		H = 2.40; *p* = 0.301	H = 6.52; *p* = 0.038 *	H = 2.81; *p* = 0.245	H = 8.80; *p* = 0.012 *	H = 1.57; *p* = 0.456
<5	97	65.64 (14.83)		65.86 (15.35)	53.77 (16.12)	55.71 (15.05)	57.69 (14.66)	51.84 (14.72)
5–13	119	63.41 (12.22)		63.12 (11.56)	49.04 (15.34)	53.38 (13.71)	53.01 (13.56)	50.49 (13.65)
>13	28	66.77 (11.38)		65.74 (11.56)	49.29 (10.73)	56.57 (11.91)	51.86 (11.70)	52.71 (10.16)
Work-shift pattern		H = 9.34; *p* = 0.025 *		H = 9.28; *p* = 0.026 *	H = 32.05; *p* ≤ 0.001 *	H = 10.54; *p* = 0.015 *	H = 17.78; *p* ≤ 0.001 *	H = 6.84; *p* = 0.077
Day	37	62.82 (13.71)	64.56 (14.41)	49.30 (14.91)	55.78 (13.23)	54.16 (13.52)	50.49 (11.68)	
Morning regular	16	67.80 (17.35)	68.52 (16.84)	53.25 (19.03)	54.75 (18.11)	56.75 (15.68)	50.75 (21.25)	
Night	79	61.53 (9.30)	61.09 (9.20)	43.90 (9.61)	50.53 (10.78)	49.72 (9.64)	48.81 (9.64)	
Rotating (both)	112	67.07 (14.37)	66.34 (14.25)	56.14 (16.26)	57.21 (15.23)	58.18 (15.47)	53.36 (15.25)	

Note: Values are mean (SD) on the transformed 0–100 metric. *n*, number of participants; SD, standard deviation. Test statistics: t, independent-samples *t*-test; F, one-way ANOVA; H, Kruskal–Wallis test. Post-hoc testing used Tukey’s HSD for job title and Dunn’s test with Bonferroni adjustment for age group, years of ICU experience, and work-shift pattern. For marital status, Holm–Bonferroni adjustment across the six domains rendered the work-performance comparison non-significant (adjusted *p* = 0.24). * *p* < 0.05.

**Table 3:** Multivariable linear regression models for perceived noise and effect scores (*n* = 244).

Predictor	Internal Noise	External Noise	Subjective	Emotional	Physiological	Work Performance
Gender (ref: male)	*p* = 0.140	*p* = 0.036 *	*p* = 0.008 *	*p* = 0.009 *	*p* = 0.081	*p* = 0.179
Female (vs. male)	2.66 (−0.86 to 6.18); β = 0.09; *p* = 0.139	3.62 (0.26 to 6.98); β = 0.13; *p* = 0.035 *	4.69 (1.24 to 8.14); β = 0.14; *p* = 0.008 *	4.72 (1.19 to 8.25); β = 0.15; *p* = 0.009 *	2.77 (−0.33 to 5.88); β = 0.09; *p* = 0.080	2.39 (−1.09 to 5.87); β = 0.08; *p* = 0.178
Age group (ref: 18–25 years)	*p* = 0.617	*p* = 0.665	*p* = 0.263	*p* = 0.060	*p* = 0.151	*p* = 0.540
26–35 years	2.28 (−3.23 to 7.80); β = 0.09; *p* = 0.417	2.78 (−2.59 to 8.16); β = 0.10; *p* = 0.310	1.24 (−3.66 to 6.14); β = 0.04; *p* = 0.620	0.06 (−5.46 to 5.58); β = 0.00; *p* = 0.984	−1.04 (−5.24 to 3.16); β = −0.04; *p* = 0.628	0.49 (−4.27 to 5.24); β = 0.02; *p* = 0.841
36–45 years	0.12 (−6.25 to 6.49); β = 0.00; *p* = 0.971	1.64 (−4.48 to 7.76); β = 0.06; *p* = 0.599	−2.61 (−8.13 to 2.91); β = −0.08; *p* = 0.355	−2.51 (−8.49 to 3.48); β = −0.08; *p* = 0.411	−4.91 (−10.13 to 0.31); β = −0.16; *p* = 0.065	1.89 (−3.45 to 7.24); β = 0.06; *p* = 0.488
≥46 years	4.65 (−10.01 to 19.31); β = 0.06; *p* = 0.534	4.82 (−5.59 to 15.22); β = 0.06; *p* = 0.364	3.16 (−16.08 to 22.39); β = 0.03; *p* = 0.748	11.68 (−0.41 to 23.78); β = 0.14; *p* = 0.058	3.96 (−8.78 to 16.70); β = 0.05; *p* = 0.543	11.64 (−5.02 to 28.30); β = 0.14; *p* = 0.171
Marital status (ref: single)	*p* = 0.208	*p* = 0.104	*p* = 0.099	*p* = 0.152	*p* = 0.041 *	*p* = 0.143
Married (vs single)	2.52 (−1.39 to 6.43); β = 0.10; *p* = 0.207	3.20 (−0.65 to 7.06); β = 0.12; *p* = 0.103	3.38 (−0.62 to 7.39); β = 0.11; *p* = 0.097	2.99 (−1.09 to 7.06); β = 0.11; *p* = 0.150	4.11 (0.19 to 8.02); β = 0.15; *p* = 0.040 *	2.95 (−0.99 to 6.89); β = 0.11; *p* = 0.142
Job title (ref: charge nurse)	*p* = 0.019 *	*p* = 0.209	*p* = 0.002 *	*p* = 0.784	*p* = 0.061	*p* = 0.214
Head nurse	1.95 (−1.98 to 5.88); β = 0.06; *p* = 0.330	1.51 (−1.96 to 4.99); β = 0.05; *p* = 0.393	−0.65 (−4.82 to 3.53); β = −0.02; *p* = 0.762	−0.21 (−4.54 to 4.12); β = −0.01; *p* = 0.924	2.26 (−1.98 to 6.49); β = 0.07; *p* = 0.296	1.37 (−3.04 to 5.78); β = 0.04; *p* = 0.542
Staff nurse	6.63 (2.04 to 11.23); β = 0.25; *p* = 0.005 *	4.15 (−0.51 to 8.82); β = 0.16; *p* = 0.081	8.35 (3.10 to 13.60); β = 0.27; *p* = 0.002 *	1.49 (−3.29 to 6.26); β = 0.05; *p* = 0.542	5.89 (1.01 to 10.76); β = 0.21; *p* = 0.018 *	−3.37 (−8.09 to 1.36); β = −0.12; *p* = 0.163
ICU experience (ref: <5 years)	*p* = 0.543	*p* = 0.674	*p* = 0.998	*p* = 0.776	*p* = 0.515	*p* = 0.356
5–13 years	0.39 (−3.44 to 4.21); β = 0.01; *p* = 0.842	−1.35 (−5.36 to 2.66); β = −0.05; *p* = 0.510	−0.12 (−4.19 to 3.95); β = −0.00; *p* = 0.953	−0.68 (−4.74 to 3.39); β = −0.02; *p* = 0.744	−1.80 (−5.98 to 2.38); β = −0.06; *p* = 0.398	−3.01 (−7.19 to 1.16); β = −0.11; *p* = 0.157
>13 years	3.20 (−2.76 to 9.16); β = 0.08; *p* = 0.293	0.44 (−4.63 to 5.50); β = 0.01; *p* = 0.866	−0.16 (−6.12 to 5.80); β = −0.00; *p* = 0.958	1.27 (−4.66 to 7.21); β = 0.03; *p* = 0.674	−3.47 (−9.71 to 2.77); β = −0.08; *p* = 0.276	−2.63 (−8.27 to 3.01); β = −0.06; *p* = 0.361
Work shift (ref: night shift)	*p* = 0.748	*p* = 0.569	*p* = 0.090	*p* = 0.156	*p* = 0.110	*p* = 0.057
Day shift	−0.83 (−5.86 to 4.20); β = −0.02; *p* = 0.747	1.95 (−3.20 to 7.09); β = 0.05; *p* = 0.458	2.37 (−2.82 to 7.57); β = 0.06; *p* = 0.371	4.35 (−0.81 to 9.51); β = 0.11; *p* = 0.099	2.35 (−2.54 to 7.23); β = 0.06; *p* = 0.347	2.82 (−1.78 to 7.43); β = 0.07; *p* = 0.229
Morning regular	3.37 (−5.35 to 12.08); β = 0.06; *p* = 0.449	5.04 (−3.50 to 13.58); β = 0.09; *p* = 0.248	6.32 (−3.84 to 16.49); β = 0.10; *p* = 0.223	0.56 (−9.04 to 10.15); β = 0.01; *p* = 0.910	4.91 (−4.13 to 13.96); β = 0.09; *p* = 0.287	0.13 (−10.20 to 10.47); β = 0.00; *p* = 0.980
Rotating (both)	1.44 (−2.23 to 5.11); β = 0.05; *p* = 0.443	1.83 (−2.16 to 5.82); β = 0.07; *p* = 0.368	5.97 (1.25 to 10.69); β = 0.19; *p* = 0.013 *	4.30 (0.05 to 8.55); β = 0.15; *p* = 0.047 *	4.72 (0.51 to 8.93); β = 0.17; *p* = 0.028 *	5.65 (1.52 to 9.77); β = 0.21; *p* = 0.007 *
Model R^2^	0.113	0.100	0.243	0.111	0.166	0.074
Adjusted R^2^	0.067	0.053	0.204	0.064	0.123	0.026
Model F (12, 231); *p*	2.83; *p* = 0.001	2.46; *p* = 0.005	6.84; *p* ≤ 0.001	3.15; *p* ≤ 0.001	3.86; *p* ≤ 0.001	1.63; *p* = 0.083

Note: Each column is a separate multivariable linear regression model containing all listed predictors simultaneously. Cells report the unstandardised coefficient B (95% confidence interval), the standardised coefficient β, and the *p* value; B is expressed in points on the transformed 0–100 subscale metric. Shaded rows give the Wald-test *p* value for the joint contribution of each multi-category predictor. Heteroscedasticity-consistent (HC3) standard errors were used throughout. * *p* < 0.05.

## Data Availability

The data presented in this study are available on reasonable request from the corresponding author. The data are not publicly available due to privacy and ethical restrictions.

## Supplementary Materials

The following supporting information can be downloaded at: https://www.mdpi.com/article/10.3390/nursrep16090342/s1. The File S1: The ICU Noise Sources and Effects Questionnaire [6].

## Public Involvement Statement

No public involvement in any aspect of this research.

## Guidelines and Standards Statement

This manuscript was drafted in accordance with the STROBE (Strengthening the Reporting of Observational Studies in Epidemiology) guidelines for cross-sectional research.

## Abbreviations

The following abbreviations are used in this manuscript:

dBA	A-weighted decibels
IBM	International Business Machines (SPSS vendor)
ICU	Intensive Care Unit
IRB	Institutional Review Board
PSMCHS	Prince Sultan Military College of Health Sciences
SPSS	Statistical Package for the Social Sciences
STROBE	Strengthening the Reporting of Observational Studies in Epidemiology
