# Supplementary material for: Perceived Noise Levels in Intensive Care Units and Their Self-Reported Adverse Effects on Nurses in Saudi Arabia: A Cross-Sectional Study
Source: Nurs Rep. 2026 Sep 19;16(9):342. doi: 10.3390/nursrep16090342 (PMC13610252; doi:10.3390/nursrep16090342)
Supplement: Supplementary file 1 [file nursrep-16-00342-s001.zip › nursrep-4512623-supplementary.pdf]

The ICU Noise Sources and Effects Questionnaire

Kooshanfar, Z., Ashrafi, S., Paryad, E., Salmanghasem, Y., Khaleghdoost Mohammadi, T., Kazemnezhad Leili, E., & Golhosseini, S. M. J. (2022). Sources of noise and their effects on nurses in intensive care units: A cross-sectional study. International Journal of Africa Nursing Sciences, 16, Article 100403. https://doi.org/10.1016/j.ijans.2022.100403

Section A: Demographic Information

Gender: ☐ Male ☐ Female

Age: ☐ 18 – 25 ☐ 26 – 35 ☐ 36 – 45 ☐ ≥ 46

Marital status: ☐ Single ☐ Married

Educational level: ☐ Diploma ☐ Bachelor ☐ Master's

ICU type: ……………………………

Number of beds: …………………………

Job title: ☐ Head nurse ☐ Staff nurse

Total years of nursing experience: ☐ <5 ☐ 5 – 14 ☐ 15 – 24 ☐ ≥ 25

Years of ICU experience: ☐ <5 ☐ 5 – 13 ☐ >13

Work shift pattern: ☐ Day shift ☐ Night shift ☐ Rotating both

Average monthly overtime: ☐ <10 hours ☐ ≥ 10 hours

Section B1: Internal ICU Noise Sources (1 = Little, 2 = Moderate, 3 = Much)

Instructions: Indicate how frequently or intensely you experience each type of noise in your ICU. Scale: 1 = Little | 2 = Moderate | 3 = Much.

Internal ICU noise source	1	2	3
1- Monitor and ventilator alarms			
2- Telephone ringing			

The ICU Noise Sources and Effects Questionnaire

Kooshanfar, Z., Ashrafi, S., Paryad, E., Salmanghasem, Y., Khaleghdoost Mohammadi, T., Kazemnezhad Leili, E., & Golhosseini, S. M. J. (2022). Sources of noise and their effects on nurses in intensive care units: A cross-sectional study. International Journal of Africa Nursing Sciences, 16, Article 100403. https://doi.org/10.1016/j.ijans.2022.100403

3- Patient expressing pain			
4- Conversation between health care staff			
5- Oxygen and suction devices			
6- Medical equipment			
7- Conversation between visitors/family members			
8- Opening and closing doors			
9- Radio or television sounds			
10- Opening drawers/wardrobes			
11- Room cleaning activities			

Section B2: External ICU Noise Sources (1 = Little, 2 = Moderate, 3 = Much)

Instructions: Indicate how frequently or intensely you experience each type of noise in your ICU.

Scale: 1 = Little | 2 = Moderate | 3 = Much.

External ICU noise source	1	2	3
1- Hospital renovation/construction			
2- Patient admissions			
3- Patients expressing pain			
4- Conversation between health care staff			

The ICU Noise Sources and Effects Questionnaire

Kooshanfar, Z., Ashrafi, S., Paryad, E., Salmanghasem, Y., Khaleghdoost Mohammadi, T., Kazemnezhad Leili, E., & Golhosseini, S. M. J. (2022). Sources of noise and their effects on nurses in intensive care units: A cross-sectional study. International Journal of Africa Nursing Sciences, 16, Article 100403. https://doi.org/10.1016/j.ijans.2022.100403

5- Conversation between visitors/family			
6- Rolling trolley wheels			
7- Opening and closing doors			
8- Hospital cleaning activities			
9- Radio or television sounds			

Section C: Effects of Noise (1 = Never – 5 = Always)

Instructions: Indicate how frequently or intensely you experience each type of noise in your ICU .

Scale: 1 = Never | 2 = Rarely | 3 = Sometimes | 4 = Often | 5 = Always

Subjective effects:

Subjective Effects	1	2	3	4	5
Anxiety					
Inattention					
Insomnia					
Fear					
Dizziness					

Emotional effects:

Emotional Effects	1	2	3	4	5
Communication problems					

The ICU Noise Sources and Effects Questionnaire

Kooshanfar, Z., Ashrafi, S., Paryad, E., Salmanghasem, Y., Khaleghdoost Mohammadi, T., Kazemnezhad Leili, E., & Golhosseini, S. M. J. (2022). Sources of noise and their effects on nurses in intensive care units: A cross-sectional study. International Journal of Africa Nursing Sciences, 16, Article 100403. https://doi.org/10.1016/j.ijans.2022.100403

Irritability					
Tension					
Loss of patience					
Tingling sensation					

Physiological effects:

Physiological Effects	1	2	3	4	5
Tachycardia					
Tinnitus					
Headache					
Fatigue					
Loss of appetite					

Work performance effects:

Work Performance Effects	1	2	3	4	5
Reduced productivity					
Concentration problems					
Negligence					
Disobedience					
Auditory masking					

The ICU Noise Sources and Effects Questionnaire

Kooshanfar, Z., Ashrafi, S., Paryad, E., Salmanghasem, Y., Khaleghdoost Mohammadi, T., Kazemnezhad Leili, E., & Golhosseini, S. M. J. (2022). Sources of noise and their effects on nurses in intensive care units: A cross-sectional study. International Journal of Africa Nursing Sciences, 16, Article 100403. https://doi.org/10.1016/j.ijans.2022.100403
